# Risk scores as useful predictors of perioperative complications in patients with rectal cancer who received radical surgery

**DOI:** 10.1007/s10147-016-1054-1

**Published:** 2016-10-25

**Authors:** Hiroshi Miyakita, Sotaro Sadahiro, Gota Saito, Kazutake Okada, Akira Tanaka, Toshiyuki Suzuki

**Affiliations:** Department of Surgery, School of Medicine, Tokai University, 143 Shimokasuya, Isehara, Kanagawa 259-1193 Japan

**Keywords:** Rectal cancer, Complication, Anastomotic leakage, Risk score

## Abstract

**Background:**

Rectal cancer is associated with a higher rate of surgical complications. The ability to predict the risk of complications before treatment would facilitate the design of personalized treatment strategies optimally suited for each patient.

**Methods:**

We retrospectively studied 260 patients with rectal cancer who underwent radical surgery to examine the relations between complications and 5 types of risk scores.

**Results:**

Complications developed in 56 patients (21.5%). Nineteen patients had infectious complications, 16 had intestinal obstruction, and 12 had other complications. Twelve patients out of 187 patients who received low anterior resection had anastomotic leakage. Estimation of Physiologic Ability and Surgical Stress Comprehensive Risk Score (E-PASS CRS) and Neutrophil-to-lymphocyte Ratio (NLR) were significantly related to all complications, infectious complications, and anastomotic leakage. Surgical Apgar Score was significantly related to infectious complications. Prognostic Nutritional Index was significantly related to all complications and intestinal obstruction. Colorectal Physiologic and Operative Severity Score for the Enumeration of Mortality and Morbidity was significantly related to all complications, and infectious complications. A multivariate analysis showed that body-mass index, E-PASS CRS, and NLR were independent risk factors for anastomotic leakage. In particular, NLR was the only score that could be evaluated before surgery.

**Conclusions:**

Five types of risk scores were useful methods for evaluating the risks of complications in patients with rectal cancer. NLR is a score that can be evaluated before surgery and predicted the risk of anastomotic leakage, suggesting that it is useful for assessing the need for a diverting colostomy.

## Introduction

Colorectal cancer is the third most common malignant disease and ranks as the third leading cause of cancer-related death [1]. The standard treatment for colorectal cancer is surgical resection. Patients with postoperative complications have been reported to have poor long-term outcomes [2, 3]. The perioperative complications is associated with not only short-term disadvantages such as a compromised quality of life, but also with increased medical costs, delayed initiation of postoperative adjuvant chemotherapy [4], high recurrence rates [5], and poor long-term outcomes [5–7]. Rectal cancer has higher incidences of infectious complications and anastomotic leakage than colon cancer [8, 9]. In particular, lower rectal cancer is an important risk factor for anastomotic leakage. The development of anastomotic leakage has been reported to increase the rate of local recurrence [10]. The international standard treatment for rectal cancer is multidisciplinary treatment, including preoperative chemoradiotherapy [11]. Surgical procedures can be selected from a number of options, including sphincter-preserving surgery, transanal local excision, and defunctioning stomas. The ability to predict the risk of complications before treatment would most likely facilitate selection of the treatment policy optimally suited for individual patients.

To date, various risk scores have been proposed for patients to undergo elective surgery, including the Estimation of Physiologic Ability and Surgical Stress Comprehensive Risk Score (E-PASS CRS) [12], Surgical Apgar Score (SAS) [13], the Prognostic Nutritional Index (PNI) proposed by Onodera et al. [14], the Neutrophil-to-lymphocyte Ratio (NLR) [15], and the Physiological and Operative Severity Score for the Enumeration of Mortality and Morbidity (POSSUM) [16]. In addition, Colorectal-POSSUM (CR-POSSUM) [19] has been proposed for patients to undergo elective surgery for colorectal cancer. In the present study, we evaluated risk scores as a means of predicting perioperative complications in patients who underwent radical surgery for rectal cancer.

## Patients and methods

From January 2003 through December 2013, a total of 392 patients underwent radical surgery for rectal adenocarcinoma in our hospital. We excluded 131 patients who underwent emergency surgery or laparoscopic surgery and studied the remaining 260 patients. Data on these patients were retrospectively collected to estimate the incidences of complications within 30 days after surgery and to compare the value of each risk score for predicting the probability of complications.

### Risk evaluation scores

We studied the following risk evaluation scores: Estimation of Physiologic Ability and Surgical Stress Comprehensive Risk Score (E-PASS CRS) [12], Surgical Apgar Score (SAS) [13], Prognostic Nutritional Index (PNI) [14], Colorectal POSSUM (CR-POSSUM) [16], and Neutrophil-to-lymphocyte Ratio (NLR) [15]. The preoperative general condition, concomitant diseases, and complications of each patient were examined from their medical records. Surgical information, such as intraoperative vital signs and bleeding volume, was obtained from each patient’s surgical and anesthesiologic records.

E-PASS CRS was calculated as described by Haga et al. [12]. The preoperative risk score, reflecting the patient’s physiological status before surgery, the surgical stress score, reflecting the degree of surgical invasion, and the comprehensive risk score, representing the overall risk associated with preoperative risk and surgical stress, were calculated for each patient. SAS was calculated from the intraoperative bleeding volume, the minimal heart rate, and the minimal mean blood pressure, as described by Gawande et al. [13]. PNI was calculated by the following formula, proposed by Onodera et al. [14]: PNI = 10 × serum albumin level (g/dL) + 0.05 × total lymphocyte count (mm^3^). CR-POSSUM was calculated as reported by Tekkis et al. [16, 17] on the basis of the Physiological Score (PS), derived from age and the results of preoperative assessments of cardiac dysfunction, systolic blood pressure, heart rate, serum hemoglobin concentration, and urea nitrogen concentration, and the Operative Severity Score (OS), derived from surgical invasion, Duke’s classification, and intraoperative findings. The CR-POSSUM score was the sum of PS and OS. NLR was calculated using blood samples obtained at initial presentation. In patients who received preoperative chemoradiotherapy, the score was calculated before chemoradiotherapy.

### Classification and severity of complications

We studied the following 4 types of complications occurring within 30 days after surgery: all complications, infectious complications (wound infection, inflammation of the pelvic dead space, and intraabdominal abscess), anastomotic leakage, and intestinal obstruction. All complications included exacerbation of underlying disease. The diagnosis of anastomotic leakage was based on the properties of drainage fluid or radiographic findings. The severity of complications was evaluated according to the Clavien-Dindo classification [18]. All complications, infectious complications, and intestinal obstruction of Clavien-Dindo grade 3a or higher that required surgical intervention and anastomotic leakage of Clavien-Dindo grade 3b or higher that required reoperation were defined as complications.

### Evaluation of risk factors for anastomotic leakage

To investigate the risk factors for anastomotic leakage, we excluded 76 patients with a diverting stoma at initial surgery from the 187 patients who underwent low anterior resection and studied the remaining 111 patients. To compare the accuracy of each score for predicting the risk of anastomotic leakage, we calculated the sensitivity, specificity, positive predictive value (PPV), negative predictive value (NPV), and accuracy rate for each score. We then compared the values among the different scores. A multivariate analysis was then performed to identify risk factors for anastomotic leakage in patients without a diverting stoma at initial surgery. The model included factors that were significantly related to anastomotic leakage in our study, as well as sex, body-mass index (BMI), smoking history, the American Society of Anesthesiologists (ASA) classification, tumor location, and preoperative chemoradiotherapy, ypStage, which have been reported to be risk factors for anastomotic leakage in patients with rectal cancer [19, 20].

### Statistical analysis

The cutoff value (COV) for each score was calculated by risk evaluation analysis, performed using receiver operating characteristic curves (ROC) in which the presence of complications was considered a positive result. The patients were divided into 2 groups according to whether their score was less than the COV or equal to or greater than the COV, and the incidence of complications was compared. For risk evaluation analysis, the COV of the PNI was set at 40, as recommended by Onodera et al. [14]. The 2 groups were compared with the use of the Chi square test. Multiple logistic regression analysis was performed. *P* values of less than 0.05 were considered to indicate statistical significance. All statistical analyses were performed with the use of JMP^®^ 10 software (SAS Institute Inc., Cary, NC, USA).

This study was approved by the institutional review board of Tokai University (15R-217).

## Results

The patients’ characteristics are shown in Table 1. The surgical procedure was lower anterior resection (LAR) in 187 patients and abdominoperineal resection (APR) in 73 patients. A total of 202 patients (77%) received preoperative chemoradiotherapy.

**Table 1:** Patients’ characteristics

Variable	*n* (%)
Sex	
Male	190 (73)
Female	70 (28)
Age (year)	
Range	28–92
Median	63
Location of the tumor	
Upper and middle	112 (43)
Lower	148 (57)
Neoadjuvant CRT	
Yes	202 (77)
No	58 (23)
Concurrent chemotherapy	
UFT	37 (19)
S-1	165 (81)
Radiation	
40 or 45 Gy	183 (91)
20 Gy and IOR	19 (9)
Surgical procedure	
LAR	187 (70)
APR	73 (30)
Histological type	
Well	129 (50)
Moderate	92 (35)
Poor	1 (0.3)
Mucinous	12 (5)
pCR	26 (10)
Lymphatic invasion	
Positive	121 (46)
Negative	138 (53)
Unknown	1 (0.3)
Venous invasion	
Positive	126 (48)
Negative	133 (51)
Unknown	1 (0.3)
Pathological stage (include ypStage)	
0	29 (11)
I	79 (30)
II	78 (31)
III	73 (28)

*CRT* chemoradiotherapy; *LAR*: low anterior resection; *APR* abdominoperineal resection; *pCR* pathological complete response

We used preoperative chemoradiotherapy for patients with clinical Stage II or III locally advanced rectal adenocarcinoma according to the NCCN guideline [11]. Tumor location was defined according to the Japanese criteria. The detail was reported previously [21].

One or more complication developed in 56 patients (21.5%). Nineteen patients (7.3%) had infectious complications, 16 (6.1%) had intestinal obstruction, and 12 (4.6%) had other complications. Anastomotic leakage was occurred in 12 patients (10.8%) out of 111 patients who received low anterior resection without diverting stomas (Table 2).

**Table 2:** Postoperative complications according to the Cravien-Dindo grade

Complication	C–D grade	*n* (%)
Infectious complication	3a	18 (27.6)
	3b	1 (1.5)
	4	0 (0)
Anastomotic leakage	3a	6 (27.2)
	3b	11 (55.0)
	4	1 (5.0)
Bowel obstruction	3a	8 (26.6)
	3b	8 (26.6)
	4	0 (0)

*C–D grade* Cravien-Dindo grade

### Evaluation of risk scores and the incidences of complications (Table 3)

E-PASS CRS was significantly related to the incidences of all complications, infectious complications, and anastomotic leakage. PNI was significantly related to the incidences of all complications and intestinal obstruction. NLR was significantly related to the incidences of all complications, infectious complications, and anastomotic leakage. SAS was significantly related to the incidence of infectious complications. CR-POSSUM was significantly related to the incidences of all complications, infectious complications, and intestinal obstruction.

**Table 3:** Relation between the predictive scoring systems and the incidence of complication

Complication	Cut-off value	Incidence (%)	OR	95% CI	*p* value
E-PASS CRS					
All complication	<0.294	16/134 (11.9)	Reference		
	≥0.294	40/126 (31.7)	3.45	1.84–6.73	<0.0001
Infectious complication	<0.294	5/134 (3.7)	Reference		
	≥0.294	14/126 (11.1)	3.23	1.19–10.23	0.0202
Anastomotic leakage	<0.294	4/72 (5.5)	Reference		
	≥0.294	8/39 (20.5)	4.38	1.28–17.46	0.0183
Bowel obstruction	<0.294	5/134 (3.7)	Reference		
	≥0.294	11/126 (8.7)	2.46	0.86–8.02	0.0906
PNI					
All complication	≥41	29/179 (16.1)	Reference		
	<40	27/81 (33.3)	2.60	1.41–4.80	0.0022
Infectious complication	≥41	12/179 (6.7)	Reference		
	<40	7/81 (8.6)	1.32	0.47–3.42	0.5750
Anastomotic leakage	≥41	8/85 (9.4)	Reference		
	<40	4/26 (15.4)	1.75	0.48–6.35	0.3907
Bowel obstruction	≥41	7/179 (3.9)	Reference		
	≥40	9/81 (11.1)	3.08	1.10–8.94	0.0311
NLR					
All complication	<2.21	17/123 (13.8)	Reference		
	≥2.21	39/137 (28.5)	2.50	1.35–4.81	0.0033
Infectious complication	<2.21	4/123 (3.2)	Reference		
	≥2.21	15/137 (10.9)	3.65	1.28–13.11	0.0138
Anastomotic leakage	<2.21	10/62 (16.1)	Reference		
	≥2.21	2/49 (4.08)	4.51	1.11–30.38	0.0329
Bowel obstruction	<2.21	5/123 (4.0)	Reference		
	≥2.21	11/137 (8.0)	2.06	0.69–6.10	0.1842
Surgcal apgar score					
All complication	≥5	48/239 (20.0)	Reference		
	<5	8/21 (38.1)	2.46	0.92–6.18	0.0692
Infectious complication	≥5	14/239 (5.8)	Reference		
	<5	5/21 (23.8)	5.02	1.47–15.09	0.0119
Anastomotic leakage	≥5	12/108 (11.1)			
	<5	0/3	–	–	0.5410
Bowel obstruction	≥5	15/239 (6.3)	Reference		
	<5	1/21 (4.8)	0.74	0.09–5.94	0.7819
CR-POSSUM					
All complication	<18	18/124 (14.5)	Reference		
	≥18	38/136 (27.9)	2.26	1.22–4.29	0.0086
Infectious complication	<18	3/124 (2.4)	Reference		
	≥18	16/136 (11.8)	5.37	1.52–18.93	0.0038
Anastomotic leakage	<18	7/75 (9.3)	Reference		
	≥18	5/36 (13.8)	1.30	0.37–4.24	0.6660
Bowel obstruction	<18	5/124 (4.0)	Reference		
	≥18	11/136 (8.1)	2.09	1.73–6.81	0.1681

*OR* odds ratio; *95* *% CI* 95% confidence interval; *E-PASS CRS* Estimation of Physiologic Ability and Surgical Stress Comprehensive Risk Score; *SAS* Surgical Apgar Score; *PNI* Onodera’s prognostic nutritional index; *NLR* neutrophilic lymphocytes ratio; *CR-POSSUM* colorectal physiological and operative severity score for the enumeration of mortality and morbidity

### Evaluation of risk factors for anastomotic leakage

Univariate analysis showed that E-PASS CRS and NLR were risk factors related to anastomotic leakage (Table 4). The ASA classification is included in E-PASS CRS and was therefore excluded. A multivariate analysis was performed, including 8 variables, i.e., 6 variables that have been reported to be risk factors for suture failure in patients with rectal cancer (sex, BMI, smoking status, tumor location, pStage, and the presence or absence of preoperative chemoradiotherapy) in addition to E-PASS CRS and NLR. The results showed that E-PASS CRS (*p* = 0.0075, odds ratio = 6.85), and NLR (*p* = 0.0089, odds ratio = 8.24) were independent risk factors for anastomotic leakage (Table 5).

**Table 4:** Univariate analysis of anastomotic leakage

Variable	Patients with leakage	Patients without leakage		*p* value
Sex				
Male	11		122	
Female	1		53	0.1045
Age				
Range	43–77		28–92	
Median	64.5		63	0.5691
BMI				
≥25	4		32	
<25	8		143	0.2009
Smoking history				
No	4		86	
Yes	8		89	0.3168
Location of the tumor				
Upper or middle	7		104	
Lower	5		71	0.9404
pStage				
0	4		21	
I	3		54	
II	2		49	
III	3		51	0.2083
CRT				
No	2		40	
Yes	10		135	0.6191
E-PASS CRS				
<0.294	4		104	
≥0.294	8		71	0.0767
NLR				
<2.21	2		86	
≥2.21	10		89	0.0292

*BMI* body mass index, *CRT* chemoradiotherapy, *E-PASS CRS* Estimation of Physiologic Ability and Surgical Stress Comprehensive Risk Score, *NLR* neutrophilic lymphocytes ratio

**Table 5:** Multivariate logistic regression analysis of anastomotic leakage

	OR	95% CI	*p* value
Sex			
Female	Reference		
Male	3.66	0.60–71.08	0.1778
BMI	1.31	1.02–1.72	0.0775
Smoking history			
No	Reference		
Yes	1.76	0.47–7.72	0.4018
Location of the tumor			
Upper or middle	Reference		
Lower	1.59	0.42–6.61	0.4903
CRT			
No	Reference		
Yes	2.13	0.42–16.82	0.3808
pStage			
0	Reference		
1	5.44	0.59–60.06	0.1317
2	7.60	0.90–84.88	0.0617
3	4.92	0.61–48.42	0.1322
E-PASS CRS			
<0.294	Reference		
≥0.294	6.85	1.63–39.63	0.0075
NLR			
<2.21	Reference		
≥2.21	8.24	1.61–76.07	0.0089

*OR* odds ratio, *95* *% CI* 95% confidence interval, *BMI* body mass index, *CRT* chemoradiotherapy, *E-PASS CRS* Estimation of Physiologic Ability and Surgical Stress Comprehensive Risk Score, *NLR* neutrophilic lymphocytes ratio

The sensitivity, specificity, PPV, NPV, and accuracy rate of the 5 scores for the prediction of anastomotic leakage were calculated (Table 6). E-PASS CRS and NLR had higher PPV, NPV, and accuracy rates than the other scores.

**Table 6:** Accuracy rate of anastomotic leakage according to predictive scoring systems

Score	COV	OR	95% CI	*p* value	Accuracy rate (%)	Sensitivity (%)	Specificity (%)	PPV (%)	NPV (%)
E-PASS CRS	0.294	4.38	1.28–17.46	0.0183	68.4	66.6	68.6	20.5	94.4
PNI	40	1.75	0.48–6.35	0.3907	27.0	66.6	22.2	9.4	84.6
NLR	2.21	4.51	1.11–30.38	0.0329	51.3	83.3	47.4	16.1	95.9
SAS	5	0	–	0.5410	13.5	100	96.9	11.0	100
CR-POSSUM	18	1.30	0.37–4.24	0.6660	34.2	58.3	31.3	9.3	86.1

*COV* cut off value; *OR* odds ratio; *95* *% CI* 95% confidence interval; *PPV* positive predictive value; *NPV* negative predictive value; *E-PASS CRS* Estimation of Physiologic Ability and Surgical Stress Comprehensive Risk Score; *SAS* Surgical Apgar Score; *PNI* Onodera’s prognostic nutritional index; *NLR* neutrophilic lymphocytes ratio; *CR-POSSUM* Colorectal physiological and operative severity score for the enumeration of mortality and morbidity

## Discussion

The development of perioperative complications in patients with rectal cancer has been reported to delay the start of adjuvant chemotherapy [4], potentially leading to poor long-term outcomes [5–7]. The ability to predict the likelihood of postoperative complications before starting treatment would thus facilitate the design of personalized treatment strategies for individual patients, including the selection of surgical procedures such as diverting colostomy.

Complications following rectal cancer surgery consisted several categories, such as cardiovascular, respiratory, urinary, wound infection, intraabdominal abscess and anastomotic leakage. However, we selected infectious complications, anastomotic leakage, intestinal obstruction and overall complications in the present study.

E-PASS is a severity score quantifying general condition and surgical risk. It has been reported to be related to postoperative complications and overall survival in elderly patients with colon cancer and those with gastric cancer [22, 23]. Haga et al. reported that E-PASS is useful for predicting the risk of anastomotic leakage in patients who have undergone gastrointestinal surgery [24, 25]. The blood lymphocyte count is an index of immune status that is used to calculate several scores. The PNI proposed by Onodera et al. is calculated from the serum albumin concentration and lymphocyte count and is a useful index of immune and nutritional status in patients with gastrointestinal cancer [14]. Patients with colorectal cancer and a low PNI have a poor prognosis [26]. A PNI of less than the COV of 45.5 has been reported to be an independent risk factor for serious complications, such as myocardial infarction and pulmonary embolism [27].

NLR is a useful prognostic factor in patients with colorectal cancer [15, 28]. A low NLR before surgery is related to disease-free survival and overall survival [29, 30]. NLR on postoperative day 1 is a risk factor for infectious complications [31, 32]. SAS is related to surgical outcomes and is calculated on the basis of bleeding volume, intraoperative minimal blood pressure, and minimal heart rate, and is thus simpler to use than CR-POSSUM and E-PASS. Patients with a high SAS after colectomy have a low incidence of complications after discharge within 30 days after surgery [33]. The modified Surgical Apgar Score (mSAS), which uses a different COV for intraoperative bleeding volume from the SAS, has been reported to be useful for predicting complications after gastrectomy [34].

CR-POSSUM is a modified score based on POSSUM [11], a severity score that quantifies general condition and surgical risk in patients with colorectal disease. CR-POSSUM is useful for predicting the risk of death within 30 days after surgery for colorectal cancer [17]. In our study, E-PASS CRS, SAS, PNI, NLR, and CR-POSSUM were useful methods for evaluating the risk of complications in patients who underwent radical surgery for rectal cancer. A multivariate analysis was performed including the 8 variables of E-PASS CRS and NLR, found to be significantly related to anastomotic leakage in our study, as well as sex, BMI, smoking history, tumor location, and the presence or absence of preoperative chemoradiotherapy, pStage which that have been reported to be risk factors for anastomotic leakage in patients with rectal cancer. The results showed that E-PASS CRS, and NLR were independent risk factors for anastomotic leakage. McDermott et al. reported co-morbidity is a risk factor for colorectal anastomotic leakage [19]. E-PASS CRS is calculated from factors including co-morbidity. NLR has not been reported to associate with anastomotic leakage in colorectal cancer up to now.

In addition, E-PASS CRS and NLR had higher PPV, NPV, and accuracy rates than the other scores.

All 5 evaluation scores assessed in our study can be calculated from general laboratory data and surgical course. However, E-PASS CRS, SAS, and CR-POSSUM include variables measured during surgery and therefore cannot be calculated until after surgery. Only PNI and NLR can be used to preoperatively evaluate risk. NLR was significantly related to anastomotic leakage and was an independent risk factor for anastomotic leakage. NLR was the only score for predicting the risk of anastomotic leakage that could be calculated preoperatively. There are many risk factors reported contributing to anastomotic leakage, such as anastomotic level from the anal verge, comorbidity, high ligation of the inferior mesenteric artery, male sex, and intraoperative complications [35].

The number of patients in the present study was small, therefore, further studies are in larger numbers of patients are needed. However, our results suggest that NLR can be used to predict the risk of anastomotic leakage preoperatively and may be helpful in determining the need for surgical procedures such as a diverting stoma.

## Conclusions

Five types of risk evaluation scores were useful for predicting perioperative complications in patients with rectal cancer who received radical surgery. E-PASS CRS and NLR were risk scores related to anastomotic leakage. NLR was the only score for predicting the risk of anastomotic leakage that could be calculated preoperatively, suggesting that it is useful for assessing the need for a diverting stoma.
